# The application of heterogeneous cluster grouping to reflective writing for medical humanities literature study to enhance students’ empathy, critical thinking, and reflective writing

**DOI:** 10.1186/s12909-016-0758-2

**Published:** 2016-09-02

**Authors:** Hung-Chang Liao, Ya-huei Wang

**Affiliations:** 1 Department of Health Services Administration, Chung Shan Medical University, Taichung, Taiwan; 2 Department of Applied Foreign Languages, Chung Shan Medical University, 110, Sec. 1, Jian-Koa N. Road, Taichung, 402 Taiwan; 3 Department of Medical Education, Chung Shan Medical University Hospital, Taichung, Taiwan

**Keywords:** Medical humanities literature study, Reflective writing, Heterogeneous grouping, Empathy, Critical thinking

## Abstract

**Background:**

To facilitate interdisciplinary collaboration and to make connections between patients’ diseases and their social/cultural contexts, the study examined whether the use of heterogeneous cluster grouping in reflective writing for medical humanities literature acquisition could have positive effects on medical university students in terms of empathy, critical thinking, and reflective writing.

**Methods:**

A 15-week quasi-experimental design was conducted to investigate the learning outcomes. After conducting cluster algorithms, heterogeneous learning clusters (experimental group; *n* = 43) and non-heterogeneous learning clusters (control group; *n* = 43) were derived for a medical humanities literature study. Before and after the intervention, an Empathy Scale in Patient Care (ES-PC), a critical thinking disposition assessment (CTDA-R), and a reflective writing test were administered to both groups.

**Results:**

The findings showed that on the empathy scale, significant differences in the “behavioral empathy,” “affective empathy,” and overall sections existed between the post-test mean scores of the experimental group and those of the control group, but such differences did not exist in “intelligent empathy.” Regarding critical thinking, there were significant differences in “systematicity and analyticity,” “skepticism and well-informed,” “maturity and skepticism,” and overall sections. As for reflective writing, significant differences existed in “ideas,” “voice and point of view,” “critical thinking and representation,” “depth of reflection on personal growth,” and overall sections, but not in “focus and context structure” and “language and conventions.”

**Conclusion:**

This study outlined an alternative for using heterogeneous cluster grouping in reflective writing about medical humanities literature to facilitate interdisciplinary cooperation to provide more humanizing medical care.

**Electronic supplementary material:**

The online version of this article (doi:10.1186/s12909-016-0758-2) contains supplementary material, which is available to authorized users.

## Background

Since 1960, research has shown the importance of humanities, declaring that the language of medicine has overemphasized diseases, thereby devaluing human beings and persistently neglecting patients’ social and cultural contexts. Hence, it is necessary to add humanities to medical curricula, for consideration of moral and ethical dilemmas. To develop medical university students’ sensitivity, empathy, and understanding of human conditions from a medical humanities perspective, it is necessary to incorporate humanities and art into existing curricula to balance the largely scientific content; such lessons act as a vehicle for exploring what it means to be humane [1, 2].

### Humanities and literature study

Research also shows that medical university students with backgrounds in humanities and sciences would perform better in practice than those with backgrounds in science alone [3, 4], because medical science cannot offer a complete picture of humanities. While science endeavors to create universal formulas, the plots in novels are always unique, because each individual has a different personal history. Serving as a complementary study of humanities, literature study can help foster human and humane understanding, by developing skills of observation, analysis, insight, reasoning, empathy, and self-reflection [5].

Sometimes, literary works represent the biopsychosocial condition of humans. Hence, literature can teach and can elucidate health and illness, giving new insights to the medical care professionals [6]. Medical care educators have used arts and literature in curricula to enhance medical humanities. Before beginning their medical care practice, students can use literary works exemplars of how to work with real patients and communicate professionally [2, 7]. According to Darbyshire [8], literature can serve the following functions in medical care education. First, by appreciating literature, students consider new ways to solve problems, make decisions, and balance personal and professional conflicts, and can thereby develop deeper understandings of the complexity of human experience, such as suffering, loss, and bereavement. Second, by sharing literature through dialogue and critiques, students can develop interpretive, critical, and analytical abilities. Third, students discussing literature develop a lifelong learning community.

In addition, the incorporation of humanities and art into curricula is a signal to medical university students that they should not only respond to patients’ bodies, they should also respond to patients’ feelings, minds, wills, and ethical choices [9]. Hence, in order to make a connection between patients’ diseases and their social or cultural contexts, or a connection between the fragility of human life and the limitations of medical technology, medical education should also train students to develop their self-reflective capacity.

### Reflection

Reflection can be defined as a cycle of deliberate, systematic, and structured intellectual inquiry activities, for the purpose of making sense of a troubling situation or dilemma [10]. Reflective wiring about medical care issues can help medical university students have a holistic and empathetic understanding of the human or patient experience in the context of a largely technological and scientific educational experience. By discussing and reflecting upon moral, philosophical, and social issues in literature, students can develop the ability to empathize with others, that is, to willingly subject their minds to patients’ worlds and expand their understandings of the complexity of the lives they encounter [11].

Moreover, reflective writing enhances critical thinking, organizes thought, and improves analysis and synthesis, thereby letting students actively construct knowledge for themselves and their practices. While examining different perspectives based on their professional medical care knowledge, students begin to question existing knowledge, thereby developing their judgment about medical care problems. Also, by being exposed to the dilemmas and conflicts in literature, students can view situations from multiple perspectives, thereby developing their capacity to formulate, evaluate, and defend certain issues that may occur in medical care [12]. Also, students can develop the capacity to respond to dilemmas, uncertainties, or limitations in order to make appropriate decisions [13–15]. While spending time reflecting on the medical issues in literature from multiple perspectives, students develop empathy with and respect for others [16, 17].

### Heterogeneous cluster grouping

With the increasing complexity of patients and the increased severity and chronicity of diseases, the need to rely on interdisciplinary teams to provide medical care is growing [18]. Research has shown that heterogeneous grouping can enhance students’ collaborative experiences and interactive professional relationships: compared to students in homogeneous learning groups, students in heterogeneous learning groups have more productive engagement in activities and interaction with each other [19, 20]. Grouping students based on their diverse backgrounds, disciplines, or competencies creates a heterogeneous grouping learning environment, in which students from different backgrounds can facilitate productivity in group discussions and stimulate other students to become more involved in and more responsible for their learning [21, 22]. Moreover, heterogeneously organized interdisciplinary teams can foster communication and other social skills, such as critical thinking, reflective thinking, and problem solving skills [21, 23–25]. Through interdisciplinary learning, students may understand the material in greater depth and develop the ability to synthesize, analyze, and evaluate the material [21, 24, 26].

To facilitate interdisciplinary interaction and to make connections between patients’ diseases and their social/cultural contexts, the study examined whether the use of heterogeneous cluster grouping in reflective writing about medical humanities literature could have positive effects on medical university students in terms of empathy, critical thinking, and reflective writing. To derive heterogeneous learning clusters, the cluster algorithm for heterogeneous cluster grouping was developed and tested. This algorithm was later used to examine the effect of using reflective writing in the medical humanities literature study. The following hypotheses were explored.

- *Hypothesis 1*. Students situated in heterogeneous learning clusters for reflective writing about medical humanities literature would show more empathy than those situated in non-heterogeneous learning clusters.
- *Hypothesis 2*. Students situated in heterogeneous learning clusters for reflective writing about medical humanities literature would be more inclined to use critical thinking than those situated in non-heterogeneous learning clusters.
- *Hypothesis 3*. Students situated in heterogeneous learning clusters for reflective writing about medical humanities literature would show more improvement in reflective writing performance than those situated in non-heterogeneous learning clusters.

## Methods

### Participants

To determine whether the use of reflective writing in heterogeneous cluster grouping for literature study could increase students’ empathy, critical thinking, and reflective writing, two homogeneous and normally distributed classes were used as the experimental group (43 students) and as the control group (43 students). Assignment to group was determined by coin flip. All students belonged to freshmen or juniors; therefore, they had little clinical experience. Information about the research was provided before the students agreed to participate. The participants were informed about the research purpose and were required to sign an informed consent form in accordance with the Declaration of Helsinki [27]. To ensure confidentiality, the students’ personal identities were protected and all data were identified only by numbers. However, in order to avoid the Hawthorne effect or John Henry effect, the students were not informed which groups they were in. All 86 participants (mean age = 18.32; *SD* = 0.42) were college students at colleges of medicine, medical science and technology, health care and management, and medical humanities and social sciences, with at least six years of English learning experience.

### Cluster algorithm for heterogeneous cluster grouping

The cluster algorithm for heterogeneous cluster grouping was developed as below.

- *Step 1. Normalizing the data of students*’ *scores of the empathy scale and of the critical thinking disposition assessment*

At this step, the pretest scores on the empathy scale and the critical thinking disposition assessment were collected. To avoid using different standards to measure students’ different empathy levels and critical thinking disposition levels, Eq. (1) was used to normalize the data.

1 $$ {Z}_{ij}=\frac{X_{ij}}{X_j^{\max }}, $$

where

*X*_*j*_^max^ = max{*X*_*ij*_, *i* = 1, 2, …, *n*}, *j* = 1 for the empathy scale score, 2 for the critical thinking disposition assessment score.

The notation is described as follows:

*Z*_*ij*_ is the *i*th student in the *j*th normalization score in the empathy scale/critical thinking disposition assessment.

*X*_*ij*_ is the *i*th student in the *j*th initial score in the empathy scale/critical thinking disposition assessment.

- *Step 2. Obtaining the diverse effect in each heterogeneous learning cluster*

To encourage “diverse thinking” in heterogeneous learning clusters, students should follow the following guidelines to create their heterogeneous learning clusters. First, each learning cluster should contain four to five students, including at least two females and two males. Second, each student in a learning cluster should come from a different department and college. The researchers could then compute the diverse thinking effect in each learning cluster from the normalized score in the empathy scale/the critical thinking disposition assessment. Equation (2) provides the index.

2 $$ \left|{\alpha}_1{\displaystyle \sum_{i=1}^n{\overline{Z}}_{i1}+{\alpha}_2{\displaystyle \sum_{i=1}^n{\overline{Z}}_{i2}}}\right|\le \vartheta $$

Where *α*_1_ and *α*_2_ are the fuzzy weights. *ϑ* is the threshold.

$$ {\overline{Z}}_{ij}={Z}_{ij}-{\overline{Z}}_j $$ for *j* = 1, 2.

${\overline{Z}}_j$ is the mean of the *j*th normalization score in the empathy scale/critical thinking disposition assessment.

- *Step 3. Arranging the derived heterogeneous learning clusters*

To arrange the derived heterogeneous learning clusters, the researchers computed the index of diverse effect and validated whether the index was less than or equal to the threshold. If necessary, the researchers assigned or adjusted the clusters based on the guidelines to compose their heterogeneous learning clusters.

### Cluster algorithm for non-heterogeneous cluster grouping

The cluster algorithm for non-heterogeneous cluster grouping was developed as below.

- *Step 1. Normalizing the data of students*’ *scores of the empathy scale and of the critical thinking disposition assessment*

After collecting the pretest scores on the empathy scale and the critical thinking disposition assessment, in order to avoid using different standards to measure students’ different empathy levels and critical thinking disposition levels, Eq. (3) was used to normalize the data.

3 $$ {Z}_{ij}=\frac{X_{ij}}{X_j^{\max }}, $$

where

*X*_*j*_^max^ = max{*X*_*ij*_, *i* = 1, 2, …, *n*}, *j* = 1 for the empathy scale score, 2 for the critical thinking disposition assessment score.

The notation is described as follows:

*Z*_*ij*_ is the *i*th student in the *j*th normalization score in the empathy scale/critical thinking disposition assessment.

*X*_*ij*_ is the *i*th student in the *j*th initial score in the empathy scale/critical thinking disposition assessment.

- *Step 2. Obtaining the diverse effect in each non*-*heterogeneous learning cluster*

To create the non-heterogeneous learning clusters, students should follow the following guidelines. First, each learning cluster should contain four to five students, including at least three females or three males. Second, students in each learning cluster should come from the same department or the same college, that is, they should have similar majors. The researchers could then compute the same thinking effect in each learning cluster from the normalized score in the empathy scale/the critical thinking disposition assessment. Equation (4) provides the index.

4 $$ \left|{\beta}_1{\displaystyle \sum_{i=1}^n{\overline{Z}}_{i1}+{\beta}_2{\displaystyle \sum_{i=1}^n{\overline{Z}}_{i2}}}\right|\ge \theta $$

where *β*_1_ and *β*_2_ are the fuzzy weights. *θ* is the threshold.

$$ {\overline{Z}}_{ij}={Z}_{ij}-{\overline{Z}}_j $$ for *j* = 1, 2.

${\overline{Z}}_j$ is the mean of the *j*th normalization score in the empathy scale/critical thinking disposition assessment.

- *Step 3. Arranging the derived non*-*heterogeneous learning clusters*

To arrange the derived non-heterogeneous learning clusters, the researchers computed the index of diverse effect and validated whether the index was greater than or equal to the threshold. If necessary, the researchers assigned or adjusted the clusters based on the guidelines to compose their non-heterogeneous learning clusters.

### Experimental design

The study investigated whether the use of heterogeneous cluster grouping in reflective writing about medical humanities literature could reveal any differences between the experimental group and the control group in terms of empathy, critical thinking, and reflective writing. The researchers used a quasi-experimental design in the study because in classroom research neither random selection nor random assignment of students to classes was possible. In addition, in classroom interactions, male students speak longer and more frequently than male students [28, 29]. In order to hear each gender’s voice, after expert panel discussion, in heterogeneous grouping, the number of male students was equivalent to the number of female students. Therefore, there should be at least two males or two females in order to let both male and females can speak up their opinions. However, in order to form a non-heterogeneous learning cluster, there were three males or three males per cluster. After conducting the cluster algorithms for heterogeneous and non-heterogeneous cluster grouping, heterogeneous learning clusters (experimental group) and non-heterogeneous learning clusters (control group) were derived. The students in the control group worked in non-heterogeneous cluster grouping to reflective writing for medical humanities literature study, while the students in the experimental group worked in heterogeneous cluster grouping. Therefore, the post-experimental difference could be attributed to the intervention of heterogeneous cluster grouping.

Before the experiment, an Empathy Scale in Patient Care (ES-PC), a critical thinking disposition assessment (CTDA-R), and a reflective writing test were administered to both groups to collect, analyze, and compare the test results. The pretest results showed that both groups were homogeneous in empathy, critical thinking disposition, and reflective writing (see Tables 1, 3, and 5). In the experiment, one researcher taught both the control group and the experimental group. In order to prevent coercion and experimenter bias, the researchers did not get involved in the survey data collection. One well-trained research assistant was responsible for collecting the survey data.

**Table 1:** F-test for the homogeneity of regression slope assumption for Group*the empathy pre-test

Scale	Group	Pre-test		Group*scale (interaction effect)	Homogeneity testing	
		Mean	*S.D.*			
					*F*	*p*-value
Overall	Experimental Control	139.76	24.69	Group*Overall	0.054	0.817
		139.65	24.61			
Behavior empathy	Experimental Control	56.78	9.85	Group* behavior empathy	0.175	0.677
		58.03	10.58			
Affective empathy	Experimental Control	40.89	10.23	Group* affective empathy	0.005	0.944
		39.12	10.54			
Intellectual empathy	Experimental Control	42.10	8.00	Group* intellectual empathy	0.120	0.730
		42.50	7.66			

Experimental group: *N* = 43; comparison group: *N* = 43

*S.D.* standard deviation

Both groups of students could read the literature they preferred, along with the requested teaching content and discussion topics (shown in Additional file 1). The learning activities included independent study, discussion forums, reflective writing, class presentations, and so on. The treatment lasted 15 weeks, with classes occurring two sessions per week, plus at least two hours of e-learning, in which discussion forum was facilitated. After reading medical humanities literature, including short stories, novels, and film literature, both groups went to the Medical Humanities and English Learning website (as Additional file 2) to post their reflections about troubling medical care situations or moral/ethical dilemmas in the lecture topics or the material they read. They were required to write a reflection per week, through which they responded for or provided reasons for the actions taken, using critical thinking and justifications.

The discussion forum served as a collaborative learning tool, by which, through discussion and writing, students who were less reflective could get involved in the discussion process, thus learning better writing and communication. Although monitoring the discussion, the instructor did not participate in the discussion. Each student was the facilitator of his/her learning cluster to critique and discuss ideas with one another in the forum. In 15-min class presentations, students were randomly selected to share their ideas, thoughts, and feelings with their peers. After intervention, the students were compared in the post-test results of the empathy scale, critical thinking disposition assessment, and reflective writing to evaluate their learning performances.

### Instrumentation

#### Empathy Scale in Patient Care (ES-PC)

In order to measure students’ levels of empathy, the researchers developed the Empathy Scale in Patient Care (ES-PC; see Additional file 3) to test students’ empathy awareness. After exploratory factor analysis, the researchers extracted 23 items and three factors, based on a nine-point rating scale, with nine meaning “strongly agree” and one meaning “strongly disagree.” The three factors were: behavioral empathy (nine items), affective empathy (seven items), and intellectual empathy (seven items). The higher the score, the more importance a student places on the issues related to empathy presented in the survey. The Cronbach’s alpha values for the three subscales were 0.93, 0.87, and 0.88, and the Cronbach alpha for the entire questionnaire was 0.94. The test-retest reliability of the final version of the scale was 0.89.

#### Critical Thinking Disposition Assessment (CTDA-R)

Yuan, et al’s [30] nine-point Likert scale of critical thinking disposition assessment (CTDA-R; see Additional file 4) was used to measure the participants’ levels of critical thinking disposition, ranging from 9 (strongly agree) to 1 (strongly disagree). The CTDA-R included 19 items and three factors: systematicity and analyticity (eight items), inquisitiveness and conversance (six items), and maturity and skepticism (five items). The students who received higher scores on the scale are interpreted as the students having higher levels of critical thinking disposition. The Cronbach’s alpha values for the three subscales were 0.93, 0.88, and 0.88, respectively, and the Cronbach’s alpha for the entire questionnaire was 0.95. The test-retest reliability of the final version of the scale was 0.87.

#### Analytic Reflective Writing Scoring Rubric (ARWSR)

To understand the performance of reflective writing in heterogeneous cluster grouping, after an extensive literature review and expert panel discussion, an Analytical Reflective Writing Scoring Rubric (ARWSR), using 0- to 5-point rating, was designed to assess students’ reflective writing ability in focus & context structure, ideas, voice & point of view, critical thinking & representation, depth of reflection on personal growth, and language & conventions. In the analytical scoring rubric, the students’ performances were divided into essential dimensions or components and scored separately. To verify the content validity, a multidisciplinary team went through runs of panel discussion to consensually agree upon the descriptions and the psychometric properties of the rubric. Scores between 0 and 6 indicated a failure or unacceptable reflective writing; scores between 7 and 12, poor reflective writing; scores between 13 and 18, acceptable reflective writing; scores between 19 and 24, strong reflective writing; scores between 25 and 30, excellent reflective writing. Two raters independently rated seven reflective writing pre-test performances. Based on the data from the baseline (*n* = 7) ratings, the mean score was 19.81, with a maximum possible score of 30. The inter-rater reliability using Cronbach’s alpha was 0.82. In the study of 86 participants, the inter-rater reliabilities between the ratings of the first and second rater were between 0.82 and 0.88 (*p* < .001), revealing the overall agreement of the two raters.

### Data analysis

The quantitative data was analyzed using Statistical Packages for Social Science Version 14.0. ANCOVA (Analysis of Covariance) was used to compare the post-test scores, using pre-test scores as a covariate to control the probable initial group differences. The post-test results were also compared using an ANCOVA to see the effects of intervention in terms of empathy, critical thinking disposition, and reflective writing. The 95 % confidence level (*p* < 0.05) was used as the criterion level to determine the statistical significance.

## Results

- *Hypothesis 1. Students situated in heterogeneous learning clusters to for reflective writing about medical humanities literature will show more empathy than those situated in non*-*heterogeneous learning clusters*.

To test Hypothesis 1, pre- and post-test results for both groups’ empathy tests were assessed using an ANCOVA. Homogeneity of regression (slope) was first conducted to test the assumption of the interaction between the pre-test scores and the groups in the prediction of students’ post-test scores. The results (see Table 1) indicated that the interactions were insignificant (F (1, 82) = 0.054, 0.175, 0.005, and 0.120; *p* = 0.817, 0.677, 0.944, and 0.730 > 0.05) and that the regression slopes were homogenous in terms of overall “behavioral empathy,” “affective empathy,” and “intelligent empathy.” In other words, before the intervention, these two groups were on par in terms of empathy. Thus, an ANCOVA could be used for data analysis to determine the effects of intervention on empathy.

After 15 weeks of experiment, an ANCOVA was used to compare the effects of intervention on students’ empathy post-test. Table 2 shows the adjusted post-test mean scores of the overall scale and subscales between the two groups. Following the intervention, the overall scores as primary outcomes showed that there was a significant difference between the two groups at a 0.01 significance level (F(1, 83) = 12.459; *p* = 0.001 < 0.01). The mean score of the experimental group (mean = 173.97) was significantly higher than that of the control group (mean = 159.68). While investigating the adjusted post-test mean scores of the subscales, the researchers found that there was no significant difference between the adjusted post-test mean score (mean = 54.43) of the experimental group and that of the control group (means = 52.33) on the “intellectual empathy” (F(1, 83) = 1.633; *p* = 0.205 > 0.05). In the sections on “behaviour empathy” and “affective empathy”, the adjusted post-test mean scores (means = 75.52 and 44.18) of the experimental group were significantly higher than those (means = 68.59 and 38.61) of the control group at a 0.01 significance level (F(1, 83) = 13.203 and 8.010; *p* = 0.000 and 0.006 < 0.01).

**Table 2:** ANCOVA comparison of the adjusted means of empathy for the post-test

Scale	Group	Adjusted mean of post-test	*F*	*p*-value
Overall	Experimental Control	173.97	12.459	0.001**
		159.68		
Behavior empathy	Experimental Control	75.52	13.203	0.000**
		68.59		
Affective empathy	Experimental Control	44.18	8.010	0.006**
		38.61		
Intellectual empathy	Experimental Control	54.43	1.633	0.205
		52.33		

Experimental group: *N* = 43; comparison group: *N* = 43

*S.D.*, standard deviation

**p < 0.01

- *Hypothesis 2. Students situated in heterogeneous learning clusters for reflective writing about medical humanities literature will be more inclined to use critical thinking than those situated in non*-*heterogeneous learning clusters*.

To test Hypothesis 2, pre-test and post-test results for both groups’ critical thinking dispositions were examined using an ANCOVA. As shown in Table 3, the results of homogeneity of regression (slope) demonstrated that the interaction between the critical thinking disposition pre-test scores and the groups in the prediction of students’ post-test scores was insignificant (F (1. 82) = 0.000, 0.017, 0.000, and 0.001; *p* = 0.989, 0.898, 0.990, and 0.970 >0.05) and that the regression slopes were homogenous. In other words, before the intervention, these two groups were on par in terms of overall, “systematicity and analyticity,” “inquisitiveness and well-informed,” and “maturity and skepticism.” Thus, an ANCOVA could be used for data analysis to determine the effects of intervention on critical thinking.

**Table 3:** F-test for the homogeneity of regression slope assumption for Group*the critical thinking disposition pre-test

Scale	Group	Pre-test		Group*scale (interaction effect)	Homogeneity testing	
		Mean	*S.D*		*F*	*p*-value
Overall	Experimental Control	75.21	10.63	Group* Overall	0.000	0.989
		75.27	10.65			
Systematicity and analyticity	Experimental Control	30.86	4.59	Group*Systematicity and Analyticity	0.017	0.898
		30.96	4.57			
Inquisitiveness and knowledgeability	Experimental Control	23.77	3.69	Group*Inquisitiveness and knowledgeability	0.000	0.990
		23.60	3.79			
Maturity and skepticism	Experimental Control	20.58	3.96	Group* Maturity and Skepticism	0.001	0.970
		20.72	3.95			

Experimental group: *N* = 43; comparison group: *N* = 43

*S.D.* standard deviation

Table 4 shows the adjusted post-test mean scores of the overall scale and subscales between the two groups. Following the intervention, the overall scores as primary outcomes showed that there was a significant difference between the two groups at a 0.01 significance level (F(1, 83) = 17.43; *p* = 0.000 < 0.01). The overall mean score of the experimental group (mean = 102.56) was significantly higher than that of the control group (mean = 92.54).

**Table 4:** ANCOVA comparison of the adjusted means of the critical thinking disposition for the post-test

Scale	Group	Adjusted mean of post-test	*F*	*p*-value
Overall	Experimental Control	102.56	17.430	0.000**
		92.54		
Systematicity and analyticity	Experimental Control	42.84	15.158	0.000**
		38.56		
Inquisitiveness and knowledgeability	Experimental Control	31.47	9.771	0.002**
		28.98		
Maturity and skepticism	Experimental Control	28.24	23.034	0.000**
		25.00		

Experimental group: *N* = 43; control group: *N* = 43

*S.D.* standard deviation

**p < 0.01

While investigating the adjusted post-test mean scores of the subscales, the researchers found that in all of the subscales (“systematicity and analyticity,” “inquisitiveness and well-informed,” and “maturity and skepticism”), the adjusted post-test mean scores (means = 42.84, 31,47, and 28.24) of the experimental group were all significantly higher than those of the control group (means = 38.56. 28.98, and 25.00) at a 0.01 significance level (F(1, 83) = 15.158, 9.771, 23.034; *p* = 0.000, 0.002, and 0.000 < 0.01).

- *Hypothesis 3. Students situated in heterogeneous learning clusters to for reflective writing about medical humanities literature will show more improvement in reflective writing performance than those situated in non*-*heterogeneous learning clusters*.

To test Hypothesis 3, pre-test and post-test results for both groups’ reflective writing scores were examined using an ANCOVA. The results of homogeneity of regression (slope) demonstrated that the interactions between the reflective writing pre-test scores and the groups in the prediction of students’ post-test scores were insignificant (F (1. 82) = 1.798, 0.774, 0.051, 0.253, 0.044, and 0.726; *p* = 0.184, 0.382, 0.821, 0.616, 0.834, and 0.397 > 0.05; see Table 5). In other words, the two groups were homogeneous in the reflective writing subsections. Thus, an ANCOVA could be used for the data analysis to see the effects of intervention on reflective writing performance.

**Table 5:** F-test for the homogeneity of regression slope assumption for Group* the reflective writing pre-test

Proficiency	Group	Pre-test		Group* Proficiency (Interaction Effect)	Homogeneity Testing	
		Mean	*S.D.*		*F*	*p*-value
Focus & context structure	Experimental Control	3.22	0.85	Group* focus & context structure	1.798	0.184
		3.37	0.82			
Ideas	Experimental Control	4.34	0.90	Group*ideas	0.774	0.382
		3.79	0.94			
Voice & point of view	Experimental Control	3.30	0.77	Group* voice & point of view	0.051	0.821
		3.61	0.66			
Critical thinking & representation	Experimental Control	3.44	1.01	Group* critical thinking & representation	0.253	0.616
		3.19	1.14			
Depth of reflection on personal growth	Experimental Control	3.48	1.02	Group* depth of reflection on personal growth	0.044	0.834
		3.65	0.89			
Language & conventions	Experimental Control	2.95	0.62	Group* language & conventions	0.726	0.397
		3.02	0.99			

Experimental group: *N* = 43; comparison group: *N* = 43

*S.D.* standard deviation

Table 6 shows the adjusted post-test mean scores of the proficiencies. On the “focus & context structure” and “language and conventions,” there were no significant difference between the adjusted post-test mean scores (mean = 3.97 and 3.49) of the experimental group and those of the control group (means = 3.98 and 3.58; F(1, 83) = 0.005 and 0.346; *p* = 0.942 and 0.558 > 0.05). On “critical thinking and representation,” there was a significant difference between the adjusted post-test mean score (mean = 4.74) of the experimental group and that of the control group (mean = 4.28) at a 0.05 significance level (F(1, 83) = 4.157; *p* = 0.045 < 0.05). On “ideas,” “voice and point of view,” and “depth of reflection on personal growth,” there was a significant difference between the adjusted post-test mean scores (means = 5.25, 4.88, and 4.94) of the experimental group and those of the control group (means = 4.44, 4.24, and 4.27) at a 0.01 significance level (F(1, 83) = 21.256, 12.260, and 11.852; *p* = 0.000, 0.001, and 0.001 < 0.01).

**Table 6:** ANCOVA comparison of the adjusted means of the reflective writing post-test

Proficiency	Group	Adjusted mean of post-test	*F*	*p*-value
Focus & context structure	Experimental Control	3.97	0.005	0.942
		3.98		
Ideas	Experimental Control	5.25	21.256	0.000**
		4.44		
Voice & point of view	Experimental Control	4.88	12.260	0.001**
		4.24		
Critical thinking & representation	Experimental Control	4.74	4.157	0.045*
		4.28		
Depth of reflection on personal growth	Experimental Control	4.94	11.852	0.001**
		4.27		
Language & conventions	Experimental Control	3.49	0.346	0.558
		3.58		

Experimental group: *N* = 43; comparison group: *N* = 43

*S.D.* standard deviation

*p < 0.05; **p < 0.01

## Discussion

The purpose of the study was to see whether heterogeneous cluster grouping for reflective writing about medical humanities literature could have positive effects on medical university students in terms of empathy, critical thinking, and reflective writing. The results reveal that students in heterogeneous cluster groups have better awareness of empathy, stronger critical thinking dispositions, and deeper reflective thinking. In order to clearly illustrate the results, a summarized table is presented below to reveal the differences between the two groups (see Table 7).

**Table 7:** A summarized table of the differences between the experimental and control groups in empathy, critical thinking disposition, and reflective writing

Scale & subscales	Empathy			
Group comparison	Overall	Behavior empathy	Affective empathy	Intellectual empathy
ANCOVA comparison	**p < 0.01	**p < 0.01	**p < 0.01	_
Scale & subscales	Critical thinking disposition			
Group comparison	Overall	Systematicity and Analyticity	Inquisitiveness and knowledgeability	Maturity and Skepticism
ANCOVA comparison	**p < 0.01	**p < 0.01	**p < 0.01	**p < 0.01
Scale & subscales	Reflective Writing			
Group comparison	Focus & context structure	Ideas	Voice & point of view	
ANCOVA comparison	_	**p < 0.01	**p < 0.01	
Scale & subscales	Reflective Writing			
Group comparison	Critical thinking & representation	Depth of reflection on personal growth	Language & conventions	
ANCOVA comparison	**p < 0.01	**p < 0.01	_	

The data analysis shows that on the empathy scale, critical thinking scale, and reflective writing, the post-test scores of both groups are all higher than the pre-test scores. The results correspond with Charon et al.’s study [16] and Hurwitz’s study [2] that literature study can help readers perceive the psychology and relationships of characters and hence evoke certain emotional engagement and empathetic actions toward those characters. Also, through literature study, students can develop skills of observation, analysis, empathy, and self-reflection in order to have a much more humane understanding of human suffering [5].

In addition, the results also correspond with Kagan and Kagan’s study [21] and Evertson and Neal’s study [23] in that students situated in heterogeneously organized interdisciplinary teams can develop better communication and social skills because heterogeneous groupings can facilitate productivity in group discussions as students stronger in certain competency may initiate discussions and stimulate other students’ participation [22].

As for the empathy, there were significant differences in the “behavioral empathy,” “affective empathy,” and overall sections between the post-test mean scores of the experimental group and those of the control group, but not in the “intelligent empathy” category. Students in heterogeneous learning clusters had a greater chance of interacting with students from different disciplines, thereby giving students a chance to improve their capacity to understand other people’s thoughts and emotions. Therefore, there is no surprise that the heterogeneous group had significantly higher post-test scores in “behavioral empathy,” “affective empathy,” and the overall scale. However, there was no significant difference in “intelligent empathy.” “Intelligent empathy” possibly belongs to a higher level of empathy awareness—that is, it requires more time for students to learn to consciously imagine themselves in the places of others and identify with them in order to genuinely understand them. “Intelligent empathy” correlates with the ability to reconstruct the viewpoints of others and sympathize with their feelings [31, 32]. Because “intellectual empathy” requires students’ constant practice in thinking, assuming, or premising within the perspectives of others, especially those with whom they strongly disagree, “intellectual empathy” is not be easily achieved in a short time.

As for critical thinking, the students in heterogeneous groups had significantly higher scores in the “systematicity and analyticity,” “skepticism and well-informed,” “maturity and skepticism,” and overall sections. The results correspond with Kagan’s, Schmidt’s, and Everston and Neal’s studies [21, 23, 25] that showed that heterogeneously grouping students must consider diversity, allowing students to discuss issues from multiple perspectives and to raise questions, defend arguments, make inferences, and draw conclusions based on evidence they have applied, analyzed, synthesized, and evaluated. Students should also make connections between what they have learned and the discussion. In addition, by discussing issues, heterogeneously grouped students experience diverse perspectives and different cognitive styles, which facilitates higher-level questioning and critical thinking [33]. Therefore, such students are trained to be more inclined to think critically.

As for reflective writing, heterogeneously grouped students have significantly higher scores in “ideas,” “voice and point of view,” “critical thinking and representation,” “depth of reflection on personal growth,” and overall sections, but not in “focus and context structure” and “language and conventions.” “Focus and context structure” and “language conventions” could belong to the basic features of effective writing [34], and both groups of students had a high-intermediate level of English language proficiency. Therefore, both groups of students could both control grammatical conventions and compose a well-organized response in the reflective writing task.

Regarding other subsections, the results support McCombs and Miller’s study [24] that suggested that while students are working with a diverse population, they begin to understand material in greater depth, because they are working collaborately to synthesize, analyze, and evaluate material. The study results also conform to Blasco and Moreto’s study [35], which demonstrated that using reflective writing about controversial issues or dilemmas, students can raise skepticism and foster analytical and synthetic thinking from a variety of perspectives, thereby achieving personal growth and avoiding past mistakes. Also, by analyzing and synthesizing the scenarios in medical humanities literature, such as dying or suffering, during the reflective writing process, students can learn how to cope with the uncertainties of life. As Lin and Shen [13] revealed, through the use of reflective writing, students can share their opinions with others; view situations from multiple perspectives; identify dilemmas, uncertainties, and limitations while confronting controversial issues; and later develop the critical thinking capacity to justify the dilemmas, uncertainties, or limitations they encounter in order to make proper decisions.

Overall, the results suggest that heterogeneous cluster grouping is worthwhile for reflective writing about medical humanities literature, because it can help give medical university students a holistic and empathetic understanding of human and patient experiences in the context of a largely technological and scientific educational experience. Furthermore, such grouping can also help students collaborate with students with different interdisciplinary backgrounds [21]. In addition, by examining different perspectives toward controversial issues or dilemmas, students can learn to analyze, synthesize, and evaluate relationships between component parts of a relationship [13]. It is therefore not surprising that heterogeneously grouped students show more empathy, are more inclined to use critical thinking, and demonstrate more improvements in reflective writing performance than non-heterogeneously grouped students.

The quasi-experimental design was a limitation of the study, though it is a form of experimental research extensively used in social sciences. However, the quasi-experimental design is a design without random pre-selection process; therefore, students in the study could not be randomly assigned to classes.

## Conclusion

Based on the findings, several conclusions can be made. First, in the empathy scale post-test, the heterogeneously grouped students had higher scores in terms of “behavioral empathy,” “affective empathy,” and overall sections than the non-heterogeneously grouped students, but they did not have higher scores in “intelligent empathy.” Second, in the critical thinking post-test, the heterogeneously grouped students had significantly higher levels of critical thinking in terms of “systematicity and analyticity,” “skepticism and well-informed,” “maturity and skepticism,” and overall sections. Third, in regard to reflective writing, heterogeneously grouped students had more improvements in “ideas,” “voice and point of view,” “critical thinking and representation,” “depth of reflection on personal growth,” and overall sections, but not in “focus and context structure” and “language and conventions.”

This study outlined an alternative for using heterogeneous cluster grouping in reflective writing about medical humanities literature to enhance medical university students’ empathy, critical thinking dispositions, and reflective writing. With the assistance of a heterogeneous cluster grouping algorithm, the study intended to let students have a chance to listen to voices in different disciplines and from different backgrounds discuss medical issues, and further facilitated interdisciplinary cooperation to provide more humanizing medical care. The development of cluster algorithms for heterogeneous and non-heterogeneous grouping is one of the contributions in the study. In addition, the most significant contribution of the study is to verify the use of heterogeneous cluster grouping to facilitate interdisciplinary collaboration and to make connection between patients’ disease and their social/cultural contexts, using reflective writing.

## Additional files

Additional file 1: The teaching content and discussion topics. The content and topics provided for medical humanities literature study. (DOCX 18 kb) Additional file 2: Medical humanities and English learning website. The learning website for reflection practice. (DOCX 369 kb) Additional file 3: Empathy Scale in Patient Care (ES-PC). Pre- (self-report items only) and post-test (all items). (DOCX 15 kb) Additional file 4: Critical Thinking Disposition Assessment (CTDA-R). Pre- (self-report items only) and post-test (all items). http://dx.doi.org/10.2224/sbp.2014.42.2.303. (DOCX 15 kb)
