# Supplementary material for: The application of heterogeneous cluster grouping to reflective writing for medical humanities literature study to enhance students’ empathy, critical thinking, and reflective writing
Source: BMC Med Educ. 2016 Sep 2;16(1):234. doi: 10.1186/s12909-016-0758-2 (PMC5010711; doi:10.1186/s12909-016-0758-2)
Supplement: Additional file 1: — The teaching content and discussion topics. The content and topics provided for medical humanities literature study. (DOCX 18 kb) [file 12909_2016_758_MOESM1_ESM.docx]

**Supplement 1** The teaching content and discussion topics

| Week | Teaching Content and Discussion Topics |
| --- | --- |
| 1 | Introduction Topics: What Are the Medical Humanities? Reflective Writing for Medical Professional Development  Teaching Content: Arts, Humanities, and Medicine; Introduction to Medical Humanities; Literature and Medicine; How Can Arts and Humanities Help Medical Understanding, Education, and Practice?  Conflict & Dilemma: Do you think medical education should offer some courses for medical humanities and ethics? If yes, which one is much more important? Medical humanities or ethics? Why? |
| 2-4 | Keyword & Lecturing: Dying with dignity; Patient Experience; Doctor-Patient Relationship; Life Value & Dignity  Medical Humanities Literature Study:  *Wit: A play* (written by [Margaret Edson](https://en.wikipedia.org/wiki/Margaret_Edson), 2001); *Wit* (directed by [Mike Nichols](http://en.wikipedia.org/wiki/Mike_Nichols), 2001) Storyline: Vivian Bearing, a demanding and uncompromising English professor of seventeenth century poetry specializing in John Donne, is diagnosed with terminal (Stage 4) ovarian cancer. During the narration, she reflects upon the side effects of to her medical treatment and significant events in her life. Her medical team, Dr. Harvey Kelekian and Dr. Jason Posner (a former student of hers), treats her only like a research experiment. However, Bearing is eager for more caring human interaction from people who see her as a human being, not just a research object….  Conflict & Dilemma: Do you think patients have the right to stop medical treatment no matter what stage of disease or cancer they are in? Do you think the government should make a policy to stop offering active medical treatment to those with terminal cancer or incurable disease; why? If yes, what assistances should the government offer such patients? |
| 5-7 | Keyword & Lecturing: Body Subjectivity; Life Meaning; Life Value & Dignity; Identity Recognition; Patient Relatives  Medical Humanities Literature Study:  *My Sister’s Keeper* (written by [Jodi Picoult](http://www.amazon.com/Jodi-Picoult/e/B000AP7PGM/ref=sr_ntt_srch_lnk_1?qid=1444265870&sr=1-1), 2004; film adaptation directed by [Nick Cassavetes](https://en.wikipedia.org/wiki/Nick_Cassavetes), 2009) Storyline: As a teenager, Anna Fitzgerald has searched for her identity. However, her mother, Sara, has defined Anna in terms of her sister, Kate. Sara used Anna as a tool to provide platelets, bone marrow, and even stem cells to help Sara’s leukemia-stricken daughter Kate survive. Anna undergoes countless surgeries, transfusions, and shots for her older sister Kate. When her mother asks Anna to donate a kidney, to insist on her body subjectivity, Anna seeks a lawyer for medical emancipation from her mother….  Conflict & Dilemma: In your opinion, what is the definition of a good parent, a good sister, or a good person? Do you think it is morally correct to do anything, even sacrifice the rights of another, to save one child’s/someone’s life? |
| 8-10 | Keyword & Lecturing: Abortion; Pro-life versus Pro-choice; Body Subjectivity; Juvenile & Sexual Desire; Women in Society  Medical Humanities Literature Study: *Juno: The Shooting Script* (written by [Diablo Cody](https://en.wikipedia.org/wiki/Diablo_Cody), 2007); *Juno* (directed by [Jason Reitman](https://en.wikipedia.org/wiki/Jason_Reitman), 2007) Storyline: After a sexual encounter with her best friend Paulie Bleeker, Juno MacGuff, a sixteen-year-old high-school student, discovers she is pregnant. Initially she considers an abortion. However, while on the way to a local abortion clinic, she comes across a schoolmate holding a rather pathetic one-person pro-life vigil. She quickly changes her mind and decides to give birth and give the baby to an adoptive couple…. Conflict & Dilemma: Are you for or against teenage pregnancy; why or why not? Are you pro-life or pro-choice? Why? Do you think people should have the body subjectivity to decide their medical practices involving the end of life, such as euthanasia, physician-assisted suicide, etc.? |
| 11-13 | Keyword & Lecturing: Anesthetic Awareness, Unconsciousness; Medical Conspiracy; Open-Heart Surgery; Doctor-Patient Relationship  Medical Humanities Literature Study:  *Awake* (written and directed by Joby Harold, 2007**)** Storyline: Clay Beresford, a genius of finance, undergoes heart surgery while experiencing a phenomenon called “anesthetic awareness,” which leaves him awake and conscious but paralyzed throughout the heart transplantation surgery. While not fully under anesthesia, Clay can hear and feel everything that is happening. He witnesses the dialogue in the room and discovers dark secrets about his surgery….  Conflict & Dilemma: What would you do if you were the young man who is awake and conscious on an operating table, with a tube inserted into your throat and blood trickling out on your surgery incision? Is there any way for you to communicate with people at the operation room, signaling to them that something is wrong and the anesthesia has not worked? Or, would you just scream voicelessly, seeing a saw cutting your bones and the rib spreaders opening your chest? |
| 13-15 | Keyword & Lecturing: Disabilities; Physical Handicapped; Brain Tumor; [Paraplegia](https://en.wikipedia.org/wiki/Paraplegia); The Disabled & Sexual Comfort; Virginity & Instinct  Medical Humanities Literature Study:  *Come as You Are* (written by Pierre De Clercq, Mariano Vanhoof, and Asta Philpot, 2011; directed by Geoffrey Enthoven, 2011)  Storyline: Twenty-somethings Lars (with incurable brain tumor), Philip (with [paraplegia](https://en.wikipedia.org/wiki/Paraplegia)), and Jozef (almost blind) are good friends and are eager for a coming-of-age journey to end their virginity. They begin to plan a visit to a luxury [brothel](https://en.wikipedia.org/wiki/Brothel) in [Spain](https://en.wikipedia.org/wiki/Spain) that specializes in offering sexual comfort to handicapped men. To organize the brothel trip, they hire an ex-midwife as a tour guide and medical care helper on the way to Spain. Everything seems all set; however, their first visit to the brothel is a disaster as Lars gets ill….  Conflict & Dilemma: Do you think that people with disabilities have the same sexual desires and needs as everyone else? If yes, should the government offer some assistance to let people with disabilities enjoy sexual intimacy? |
