# Supplementary figures and images for: The application of heterogeneous cluster grouping to reflective writing for medical humanities literature study to enhance students’ empathy, critical thinking, and reflective writing
Source: BMC Med Educ. 2016 Sep 2;16(1):234. doi: 10.1186/s12909-016-0758-2 (PMC5010711; doi:10.1186/s12909-016-0758-2)

**Supplement 2** Medical humanities and English learning website


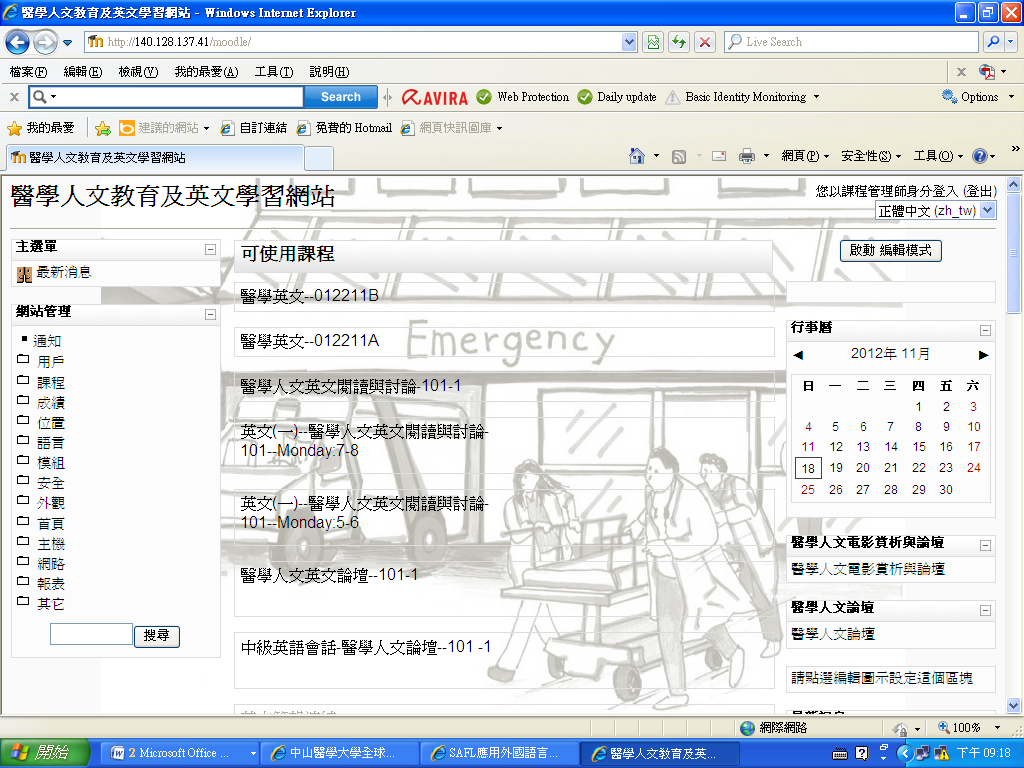

Supplement: Additional file 2: — Medical humanities and English learning website. The learning website for reflection practice. (DOCX 369 kb) [file 12909_2016_758_MOESM2_ESM.docx]
