# Supplementary material for: The application of heterogeneous cluster grouping to reflective writing for medical humanities literature study to enhance students’ empathy, critical thinking, and reflective writing
Source: BMC Med Educ. 2016 Sep 2;16(1):234. doi: 10.1186/s12909-016-0758-2 (PMC5010711; doi:10.1186/s12909-016-0758-2)
Supplement: Additional file 3: — Empathy Scale in Patient Care (ES-PC). Pre- (self-report items only) and post-test (all items). (DOCX 15 kb) [file 12909_2016_758_MOESM3_ESM.docx]

**Supplement 3** Empathy Scale in Patient Care (ES-PC) Empathy Scale in Patient Care (ES-PC)

Behavioral Empathy

1. While listening to the patient, I will face the patient and make eye contact, so that the patient may feel respected.
2. While listening to the patient, I will respond with appropriate smiles and nods.
3. When explaining the prognosis and treatment, I will express and describe things in a way that the patient may be able to understand.
4. When the patient or the patient’s family has an opinion that I disagree with or which is different than mine, I will keep my opinion to myself, wait for them to finish, and then express my opinion at an opportune time.
5. If the patient or the patient’s family has a question or argument about the prognosis or treatment, I will help them understand.
6. When the patient or the patient’s family is feeling sad because of the illness, I will comfort them.
7. When I detect that the patient or the patient’s family is feeling worried or helpless because of the illness, I will approach them and try to help reduce their worries.
8. When I find out that the patient didn’t follow the physician’s orders in taking the medication or treatment, I will restrain my negative emotion and try my best to communicate in a manner that achieves the best possible medical effectiveness.
9. When communicating with the patient or the patient’s family, I try using a gentle tone and avoid criticism.

Affective Empathy

1. When I see a patient alone and helpless, I feel sad.
2. I feel happy when the patient is happy, and I feel sad when the patient is sad.
3. When I must inform the patient of bad news, I cannot control my emotions, and I feel upset.
4. The misfortune of the patient will affect my emotions.
5. When the patient is in a distressed situation, I feel uneasy.
6. When seeing a helpless patient, I feel very upset.
7. When seeing the patient or the patient’s family crying, I also feel like crying.

Intelligent Empathy

1. I am capable of detecting the distress emotion from the patient or the patient’s family.
2. As a feeling of anger rises with the patient or the patient’s family, I will restrain myself from releasing the anger and try to put myself in their shoes.
3. Before I make a medical treatment decision, I will consider various opinions from every angle.
4. Before making a decision, I will think about it carefully from the patient’s or the patient’s family’s perspective.
5. I will try to understand the patient or the patient’s family by imagining their viewpoints.
6. I am good at interpreting the emotions of the patient or the patient’s family.
7. I am able to put myself in their perspective and appreciate the feelings of the patient or the patient’s family without any criticism.
