# Supplementary material for: The application of heterogeneous cluster grouping to reflective writing for medical humanities literature study to enhance students’ empathy, critical thinking, and reflective writing
Source: BMC Med Educ. 2016 Sep 2;16(1):234. doi: 10.1186/s12909-016-0758-2 (PMC5010711; doi:10.1186/s12909-016-0758-2)
Supplement: Additional file 4: — Critical Thinking Disposition Assessment (CTDA-R). Pre- (self-report items only) and post-test (all items). http://dx.doi.org/10.2224/sbp.2014.42.2.303. (DOCX 15 kb) [file 12909_2016_758_MOESM4_ESM.docx]

**Supplement 4** Critical Thinking Disposition Assessment (CTDA-R)

Cited from Yuan, S. P., Liao, H. C., Wang, Y., Chou, M. J. (2014). Developing a scale measuring critical thinking disposition for medical care professionals. *Social Behavior & Personality, 42*(2), 303-312. http://dx.doi.org/10.2224/sbp.2014.42.2.303.

Systematicity and analyticity

1. I am able to read between the lines, and find out any conflicting or contradictory statement in an article.
2. Before making a judgment, I am used to analyzing all the available information and the current situation.
3. I am able to determine the value of a piece of information, and then evaluate the reasonableness of the conclusion accordingly.
4. I always make sure that a piece of information is reliable before taking it into consideration.
5. I try to know every detail about controversial problems that occurred recently.
6. I draw conclusions by logical thinking and methodological analysis.
7. I always examine the pros and cons of each opinion I am exposed to.
8. I often contemplate what is right and wrong about the things I have done and experienced.

Inquisitiveness and conversance

1. I always learn as much as possible, even if I don’t know when I’ll put to use the things I learned.
2. Before making an important decision, I always make every effort to collect all the relevant information.
3. When solving a problem, I manage to keep myself updated with everything relevant.
4. I try to delve into anything, or any viewpoint, that is new and novel.
5. When making a decision, people expect me to lay down proper rules as guidance.
6. In times of trouble, I strive to seek all potential solutions before deciding on the best one.

Maturity and skepticism

1. During discussions, I am able to raise questions and respond to others’ opinions while remaining calm.
2. During discussions, I always try my best to understand, and listen to, different opinions before communicating.
3. When I am thinking, I am able to tolerate different viewpoints or opinions.
4. I will correct my viewpoint immediately when there is enough evidence to prove that it is biased.
5. I never hesitate to question any prejudice, assumption, or belief of mine and thoroughly examine everything I have said and done.
